# HIF-1α-regulated lncRNA-TUG1 promotes mitochondrial dysfunction and pyroptosis by directly binding to FUS in myocardial infarction

**DOI:** 10.1038/s41420-022-00969-8

**Published:** 2022-04-08

**Authors:** Yong-Wang Wang, Hong-Zhi Dong, Yong-Xing Tan, Xu Bao, Ying-Man Su, Xin Li, Fang Jiang, Jing Liang, Zhen-Cai Huang, Yan-Ling Ren, Yu-Li Xu, Qiang Su

**Affiliations:** 1 Department of Anesthesiology, Affiliated Hospital of Guilin Medical University, Guilin, 541001 Guangxi Zhuang Autonomous Region, P. R. China; 2 Department of Cardiology, Tianjin Chest Hospital, Tianjin, 300222 P. R. China; 3 Department of Intensive Care Unit, Affiliated Hospital of Guilin Medical University, Guilin, 541001 Guangxi Zhuang Autonomous Region, P. R. China; 4 Department of Cardiology, Affiliated Hospital of Guilin Medical University, Guilin, 541001 Guangxi Zhuang Autonomous Region, P. R. China

**Keywords:** Diseases, Cardiovascular diseases

## Abstract

Myocardial infarction (MI) is a fatal heart disease that affects millions of lives worldwide each year. This study investigated the roles of HIF-1α/lncRNA-TUG1 in mitochondrial dysfunction and pyroptosis in MI. CCK-8, DHE, lactate dehydrogenase (LDH) assays, and JC-1 staining were performed to measure proliferation, reactive oxygen species (ROS), LDH leakage, and mitochondrial damage in hypoxia/reoxygenation (H/R)-treated cardiomyocytes. Enzyme-linked immunoassay (ELISA) and flow cytometry were used to detect LDH, creatine kinase (CK), and its isoenzyme (CK-MB) levels and caspase-1 activity. Chromatin immunoprecipitation (ChIP), luciferase assay, and RNA-immunoprecipitation (RIP) were used to assess the interaction between HIF-1α, TUG1, and FUS. Quantitative real-time polymerase chain reaction (qRT-PCR), Western blotting, and immunohistochemistry were used to measure HIF-1α, TUG1 and pyroptosis-related molecules. Hematoxylin and eosin (HE), 2,3,5-triphenyltetrazolium chloride (TTC), and terminal deoxynucleotidyl transferase dUTP risk end labelling (TUNEL) staining were employed to examine the morphology, infarction area, and myocardial injury in the MI mouse model. Mitochondrial dysfunction and pyroptosis were induced in H/R-treated cardiomyocytes, accompanied by an increase in the expression of HIF-α and TUG1. HIF-1α promoted TUG1 expression by directly binding to the TUG1 promoter. TUG1 silencing inhibited H/R-induced ROS production, mitochondrial injury and the expression of the pyroptosis-related proteins NLRP3, caspase-1 and GSDMD. Additionally, H/R elevated FUS levels in cardiomyocytes, which were directly inhibited by TUG1 silencing. Fused in sarcoma (FUS) overexpression reversed the effect of TUG1 silencing on mitochondrial damage and caspase-1 activation. However, the ROS inhibitor N-acetylcysteine (NAC) promoted the protective effect of TUG1 knockdown on H/R-induced cardiomyocyte damage. The in vivo MI model showed increased infarction, myocardial injury, ROS levels and pyroptosis, which were inhibited by TUG1 silencing. HIF-1α targeting upregulated TUG1 promotes mitochondrial damage and cardiomyocyte pyroptosis by combining with FUS, thereby promoting the occurrence of MI. HIF-1α/TUG1/FUS may serve as a potential treatment target for MI.

## Introduction

As the most severe and fatal complication of ischemic heart disease, myocardial infarction (MI) causes 7.4 million deaths globally per year [1]. MI is characterized by decreased coronary blood flow which causes insufficient oxygen supply to cardiac tissue [2]. The resulting cardiac ischemia leads to mitochondrial malfunction, activation of the ischemic cascade, and cell death [3]. Treatments of the disease include early intervention by oxygen supplementation, use of anticoagulants, and immediate reperfusion [2, 3]. With the current available treatments, however, the prognosis of MI is still poor. It is therefore necessary to investigate the underlying mechanism of MI, which may help improve the understanding of the disease and potentially indicate new therapeutic targets.

Pyroptosis is a new type of programmed cell death that is characterized by the formation of membrane pores and cell lysis [4]. Pyroptosis is driven by caspase-1 or caspase-4/5/11 and has been implicated in many types of cardiovascular diseases [5]. The nucleotide oligomerization domain (NOD)-like receptor protein 3 (NLRP3) inflammasome plays essential roles in MI. NLRP3 induces cardiomyocyte pyroptosis by activating caspase-1, followed by cleavage of gasdermin D (GSDMD) and release of proinflammatory cytokines (interleukin-1β and interleukin-18) [5–9]. Some studies found that increased generation of reactive oxygen species (ROS) in mitochondrial dysfunction induced the assembly of NLRP3 and ASC at mitochondria and subsequent activation of NLRP3 [10, 11]. However, the exact role of pyroptosis in MI remains unclear.

A member of Hypoxia inducible Factor 1 (HIF-1) family, HIF-1α, was found to exert cardioprotective functions in MI [12]. However, some studies showed opposing results that HIF-1α also plays harmful roles in myocardial injuries [13–15]. Mitochondrial dysfunction was reported to decrease HIF-1α signalling in hypoxia [16], and inhibiting activation of HIF-1α prevents mitochondrial dysfunction in hepatic ischemia-reperfusion (I/R) injury [17]. Hypoxia-induced ROS activate pyroptosis of myoblasts through the HIF-1α signalling pathway[18]. These findings are, however, still quite limited, and more investigations are needed to further elucidate the relationship between HIF-1α, pyroptosis, and mitochondrial dysfunction in MI.

Long noncoding RNAs (lncRNAs) play key roles in many biological and pathological processes by binding to their regulators on chromosomes [19]. Several lncRNAs were found to be involved in myocardial I/R injury, e.g., HULC, SNHG8, UCA1, NEAT1, and TUG1 [20–23]. LncRNA-TUG1 (taurine-upregulated 1) has been well studied in cancer [24]. Previous studies by our group and other investigators suggested that TUG1 also contributed to I/R injury in MI [23, 25–27]. Our previous findings showed that the expression of TUG1 was elevated during ischemia, and silencing TUG1 expression alleviated cardiomyocyte injury *via* inhibition of ROS [27, 28]. These findings suggest key roles of TUG1 in MI, and further understanding of the detailed molecular pathways is required. Our preliminary bioinformatics analysis using JASPAR (http://jaspar.genereg.net/) found a binding site of HIF-1α on the promoter region of TUG1.

Using in vitro and in vivo MI models, our results showed for the first time that HIF-1α positively regulates TUG1 expression by binding to its promoter region, which contributes to hypoxia-induced myocardial injury in MI by promoting mitochondrial dysfunction and cardiomyocyte pyroptosis. Silencing of the HIF-1α/TUG1 pathway may be used as a potential treatment target to alleviate myocardial injury in MI.

## Results

### Mitochondrial dysfunction and pyroptosis were induced in H/R-treated cardiomyocytes

**Fig. 1:**
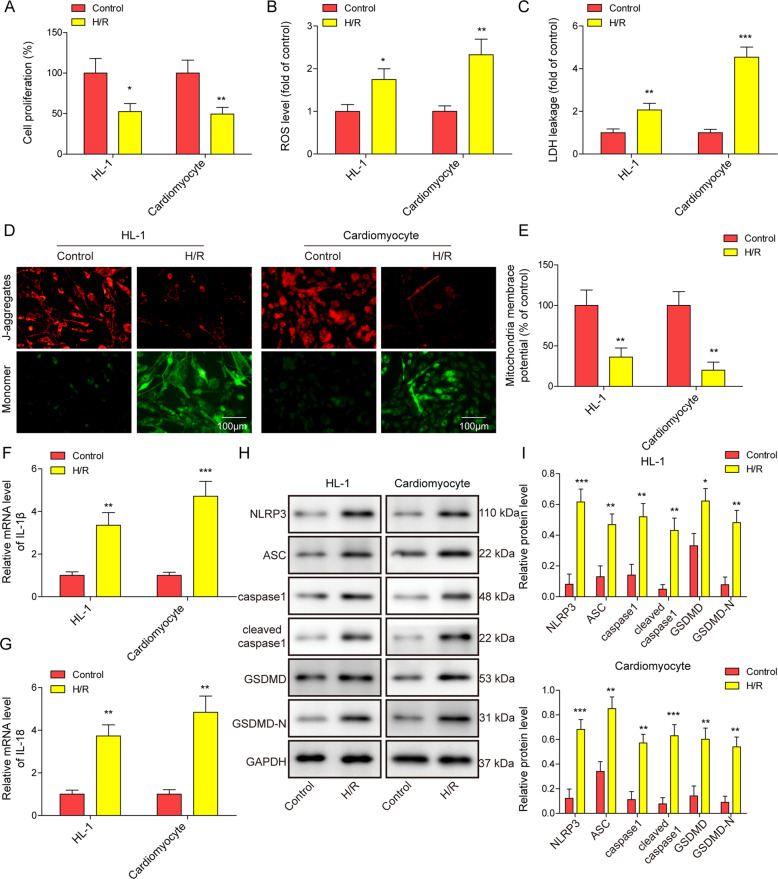
H/R treatment led to mitochondrial dysfunction and pyroptosis in cardiomyocytes. HL-1 and primary mouse cardiomyocytes were treated with H/R for 24 h. **A** Cell proliferation ability was measured using a CCK-8 assay. **B** ROS levels were measured using a DHE assay. **C** LDH levels were measured using an LDH assay kit. **D**, **E** Mitochondrial membrane potential was evaluated using JC-1 staining. **F** IL-1β and **G** IL-18 mRNA levels were assessed using qRT-PCR. **H**, **I** Western blot analysis determined the expression levels of pyroptosis-related markers. *P < 0.05, **P < 0.01 and ***P < 0.001.

We first generated in vitro hypoxic cell models by treating a mouse cardiomyocyte cell line (HL-1) and primary mouse cardiomyocytes with H/R. After H/R treatment, the proliferation ability of the cardiomyocytes was damaged (Fig. 1A), and ROS levels and lactate dehydrogenase (LDH) leakage were increased (Fig. 1B, C). JC-1 staining results showed decreased mitochondrial membrane potential (Fig. 1D, E), which indicated mitochondrial dysfunction induced by H/R treatment in cardiomyocytes. The results showed that the mRNA expression of IL-1β and IL-18 was elevated after H/R treatment (Fig. 1F, G), and Western blot analysis showed increased levels of pyroptosis-related proteins, including NLRP3, Caspase-1, ASC, GSDMD, and cleaved caspase-1, and GSDMD-N (cleaved GSDMD) (Fig. 1H, I). All these results indicated that H/R treatment induced pyroptosis in HL-1 cells and primary cardiomyocytes through activation of NLRP3 signalling.

### TUG1 expression was increased by HIF-1α in cardiomyocytes by binding to its promoter region

**Fig. 2:**
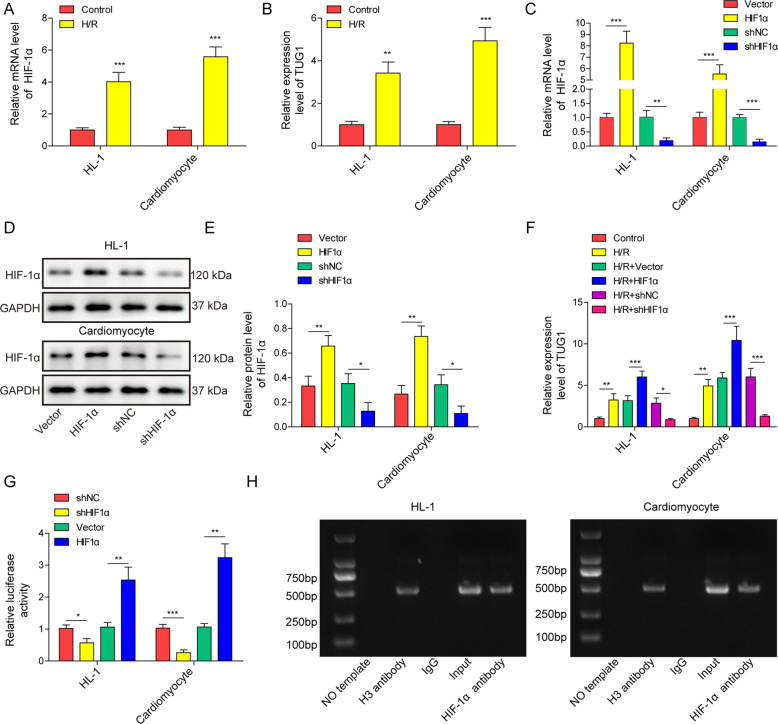
HIF-1α increased the expression of TUG1 in cardiomyocytes by binding to its promoter region. qRT-PCR measurement of (**A**) HIF-1α and (**B**) TUG1 expression in cardiomyocytes treated with H/R. **C** qRT-PCR and **D**, **E** western blot measurement of HIF-1α expression in cardiomyocytes transfected with shHIF-1α, shNC, HIF-1α, or empty vector. **F** qRT-PCR measurement of TUG1 levels in cardiomyocytes. **G** Dual luciferase reporter gene assay on the regulation of TUG1 expression by HIF-1α in cardiomyocytes. **H** ChIP assay on the presence of TUG1 after pulldown by HIF-1α antibody in cardiomyocytes. *P < 0.05, **P < 0.01 and ***P < 0.001.

Considering the potential role of HIF-1α and TUG1 in MI, we measured their expression in the H/R-treated cell models and found that the expression levels of those two factors were elevated after H/R treatment (Fig. 2A, B). After successful transfection of vector expressing HIF-1α or expressing shRNA targeting HIF-1α in both HL-1 and primary cardiomyocytes (Fig. 2C–E), the expression level of TUG1 was increased in the HIF-1α overexpression model and decreased in the HIF-1α-silenced model (Fig. 2F). Luciferase reporter assay results showed that the activity of TUG1 was enhanced when HIF-1α was overexpressed and inhibited when HIF-1α expression was silenced in cardiomyocyte models (Fig. 2G). In the chromatin immunoprecipitation (ChIP) assay, the HIF-1α antibody successfully pulled down TUG1 (Fig. 2H) in cardiomyocyte, indicating direct binding of the two factors. These results indicate that HIF-1α is an upstream regulator of TUG1 and that HIF-1α directly promotes TUG1 expression.

### H/R treatment-induced mitochondrial dysfunction and pyroptosis were inhibited by silencing TUG1 in cardiomyocytes

**Fig. 3:**
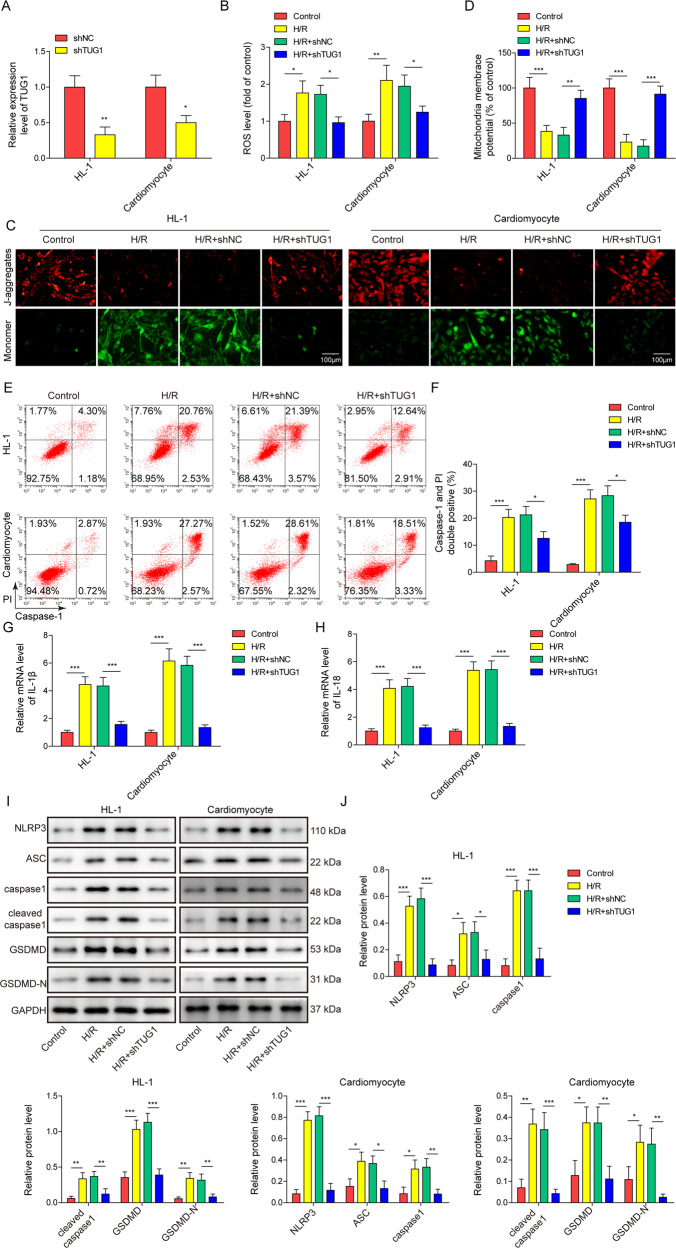
Silencing TUG1 inhibited H/R treatment-induced mitochondrial dysfunction and pyroptosis in cardiomyocytes. **A** qRT-PCR measurement of TUG1 levels in cardiomyocytes transfected with shTUG1 or cshNC. HL-1 and primary mouse cardiomyocytes were transfected with shNC or shTUG1 and then treated with H/R. **B** ROS levels were measured using a DHE assay. **C**, **D** Mitochondrial membrane potential was evaluated using JC-1 staining. **E**, **F** Caspase-1 activity was measured using flow cytometry. **G** IL-1β and **H** IL-18 expression was measured using qRT-PCR. **I**, **J** The expression of pyroptosis-related markers was measured using western blotting. *P < 0.05, **P < 0.01 and ***P < 0.001.

After silencing TUG1 expression (Fig. 3A), both HL-1 and primary mouse cardiomyocytes showed decreased ROS levels and increased mitochondrial membrane potential when they received H/R treatment (Fig. 3B–D). TUG1 silencing also inhibited the activation of pyroptosis by H/R treatment (Fig. 3E, F). Additionally, TUG1 silencing also inhibited the increase in IL-1β and IL-18 levels induced by H/R treatment (Fig. 3G, H) and the activation of pyroptosis-related proteins (Fig. 3I, J). These results further confirm that TUG1 plays key roles in H/R-induced mitochondrial dysfunction and pyroptosis of cardiomyocytes.

### TUG1 promotes FUS expression by directly binding to the protein

**Fig. 4:**
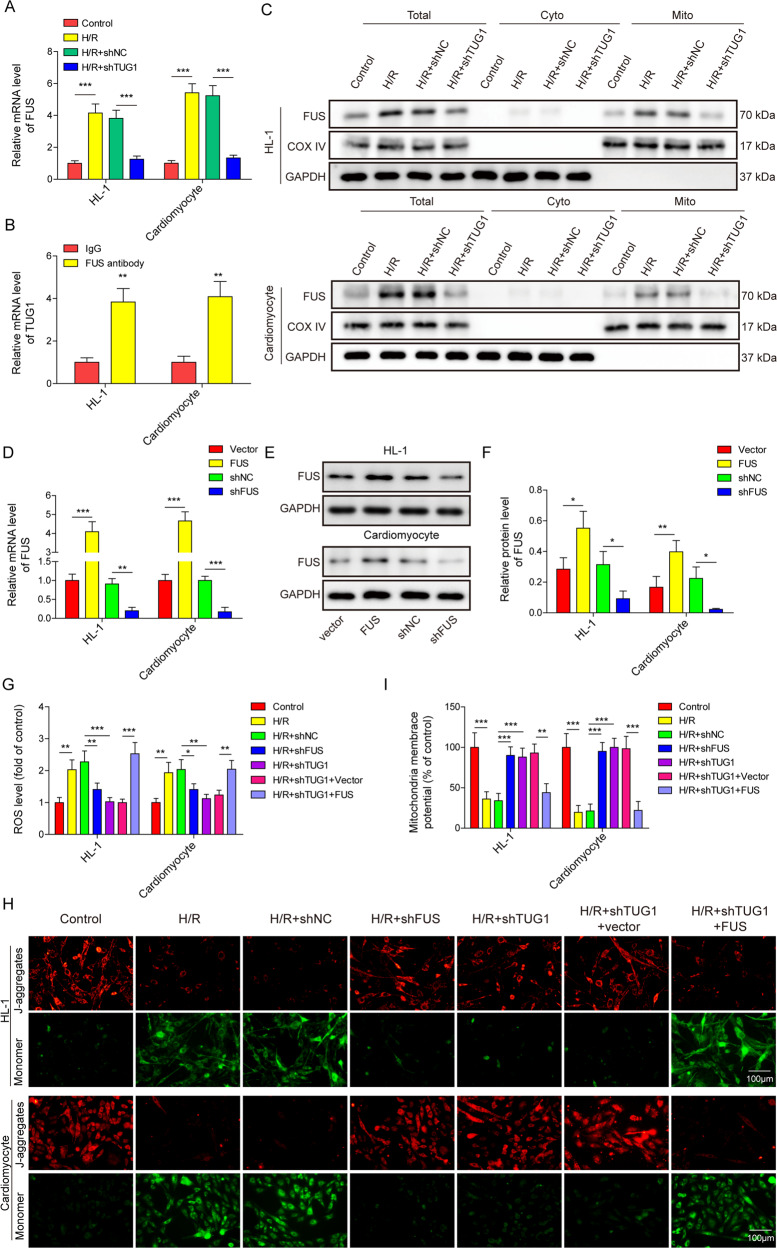
TUG1 promoted FUS expression by directly binding to the molecules, and overexpression of FUS reversed the effects of TUG1 silencing on H/R-induced mitochondrial dysfunction in cardiomyocytes. **A** qRT-PCR measurement of FUS levels in H/R-treated cardiomyocytes transfected with shTUG1 or shNC. **B** RIP assay on the presence of TUG1 in cardiomyocyte samples pulled down by antiFUS antibody or control IgG. **C** Western blotting measurement of FUS expression in the cardiomyocytes cytoplasm, mitochondrial fraction. The **D** mRNA and **E**, **F** protein levels of FUS were measured using qRT-PCR and western blotting in cardiomyocytes transfected with shFUS, shNC, vector expressing FUS, or empty vector. **G** ROS levels were measured using a DHE assay. **H**, **I** Mitochondrial membrane potential was evaluated using JC-1 staining. *P < 0.05, **P < 0.01 and ***P < 0.001.

FUS (fused in sarcoma) was reported to be involved in cardiac repair after MI [29]. The results showed that the expression of FUS was increased in H/R-treated cardiomyocytes, and silencing TUG1 inhibited FUS expression (Fig. 4A). TUG1 was pulled down using FUS antibody, indicating direct binding between TUG1 and FUS protein (Fig. 4B). Separate Western blot analysis of the proteins in the cytoplasm, mitochondrial fraction and whole cell lysates showed that H/R treatment increased FUS protein levels both in the mitochondrial fraction and whole cell samples of cardiomyocytes, while silencing TUG1 inhibited the increase in FUS observed in H/R-treated cells, which did not result in significant changes in the cytoplasm (Fig. 4C). These observations were consistent with our preliminary bioinformatics analysis results and confirmed that FUS expression is regulated by TUG1 by direct binding.

### The inhibitory effect of TUG1 silencing on H/R-induced mitochondrial dysfunction and pyroptosis was reversed by FUS overexpression in cardiomyocytes

**Fig. 5:**
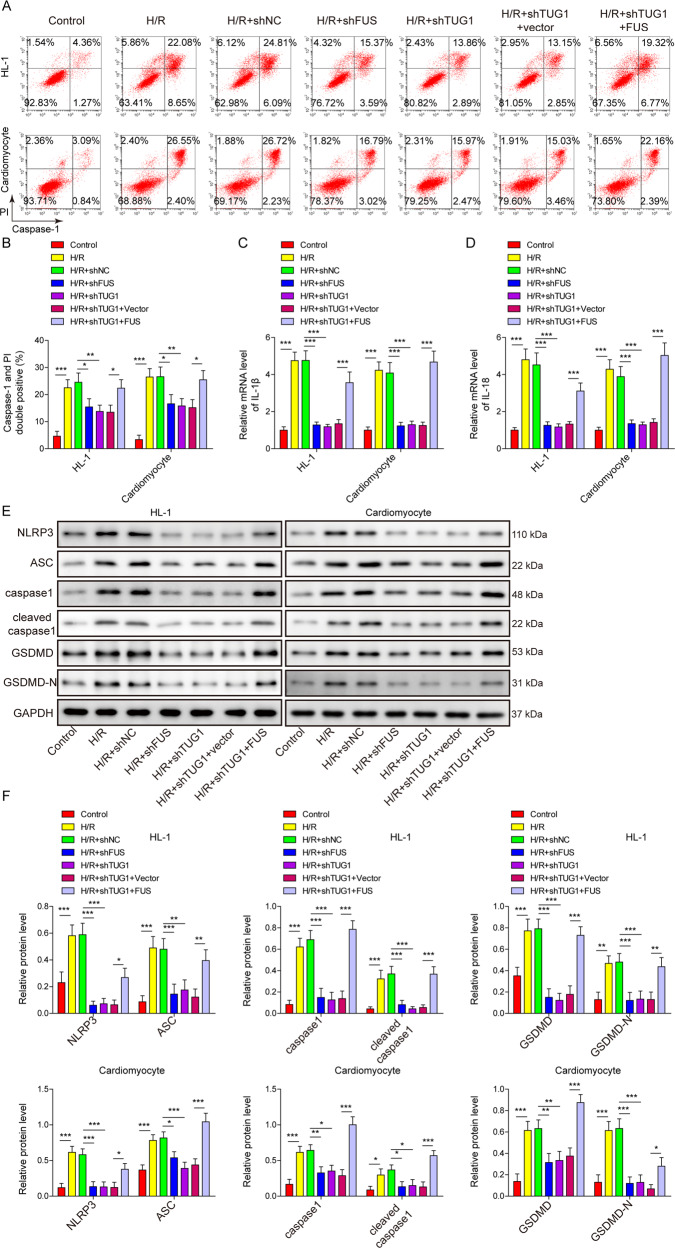
Overexpression of FUS reversed the effects of TUG1 silencing on H/R-induced pyroptosis in cardiomyocytes. **A**, **B** Caspase-1 activity was measured using flow cytometry. **C** IL-1β and **D** IL-18 expression was measured using qRT-PCR. **E**, **F** The expression of NLRP3, ASC, and other downstream pyroptosis-related factors was measured using western blotting. *P < 0.05, **P < 0.01 and ***P < 0.001.

After the generation of FUS overexpression or FUS-silenced models (Fig. 4D–F), we observed that FUS silencing inhibited the H/R-induced increase in ROS and decrease in mitochondrial membrane potential (Fig. 4G–I), as observed in TUG1 silencing. Furthermore, FUS overexpression reversed the inhibition of H/R-induced effects in TUG1-silenced cardiomyocyte models (Fig. 4G–I). We also investigated pyroptosis in our cardiomyocyte models and observed that similar to silencing TUG1, silencing FUS inhibited the H/R-induced activation of caspase-1 (Fig. 5A, B) and increased IL-1β and IL-18 expression and pyroptosis-related markers (Fig. 5C–F). Similarly, FUS overexpression also reversed the inhibition of H/R-induced changes in pyroptosis by TUG1 silencing in cardiomyocyte models (Fig. 5A–F). These results indicate that FUS is involved as the downstream signalling factor of TUG1 in H/R-induced mitochondrial dysfunction and pyroptosis in cardiomyocytes.

### H/R-induced pyroptosis was inhibited by silencing TUG1 in cardiomyocytes through inhibition of ROS production

**Fig. 6:**
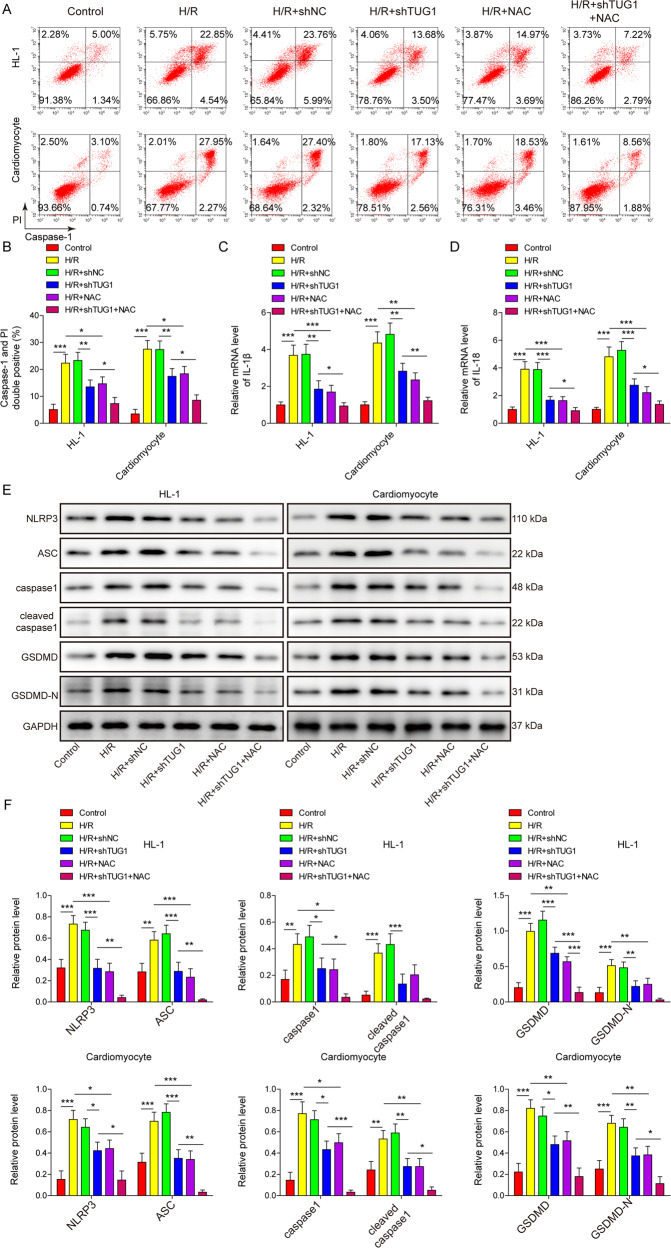
Silencing TUG1 inhibited H/R-induced pyroptosis of cardiomyocytes by decreasing the generation of ROS. HL-1 and primary mouse cardiomyocytes were transfected with shTUG1 or treated with the ROS inhibitor NAC and then subjected to H/R. **A**, **B** Caspase-1 activity was measured using flow cytometry. **C** IL-1β, and **D** IL-18 expression was measured using qRT-PCR. **E**, **F** The expression of NLRP3, ASC, and other downstream pyroptosis-related factors was measured using western blotting. *P < 0.05, **P < 0.01 and ***P < 0.001.

Several previous studies indicated that pyroptosis was inhibited by decreasing ROS levels [30–33]. Therefore, we also investigated the possible role of ROS in H/R-induced pyroptosis of cardiomyocytes. Using an ROS inhibitor, N-acetylcysteine (NAC), we observed inhibition of caspase-1 activation in H/R-treated cardiomyocytes (Fig. 6A, B). Additionally, treatment with NAC in TUG1-silenced cardiomyocytes led to even lower caspase-1 activity than treatment with NAC or TUG1 silencing alone (Fig. 6A, B). Similarly, NAC inhibited the H/R-induced increase in IL-1β and IL-18 and the expression of pyroptosis-related proteins, including NLRP3, caspase-1, ASC, GSDMD, cleaved caspase-1, and cleaved GSDMD (Fig. 6C–F), and cotreatment with NAC in TUG1-silenced cardiomyocytes showed lower levels of those factors than NAC treatment or TUG1 silencing alone. These results indicate that ROS play key roles in promoting pyroptosis in H/R-treated cardiomyocytes.

### I/R treatment-induced myocardial damage was reduced by silencing TUG1 in an in vivo mouse model

**Fig. 7:**
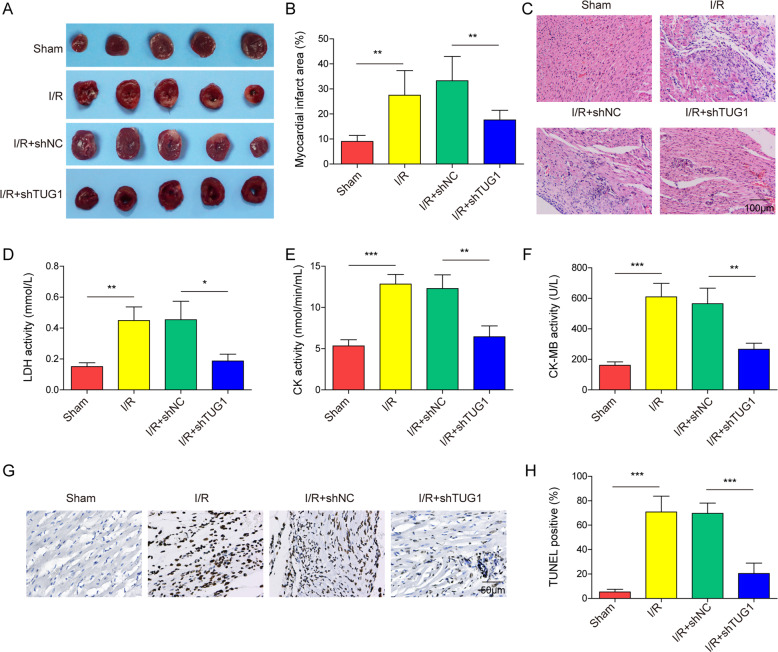
Silencing TUG1 reduced I/R-induced myocardial damage in a mouse model. **A**, **B** TTC staining was used to show the region of MI in the myocardial tissue of I/R mice. **C** Histological changes in the myocardial tissue were examined using H&E staining. The serum levels of **D** LDH, **E** CK, and **F** CK-MB were determined using ELISA. **G**, **H** Cell death was determined using TUNEL staining. *P < 0.05, **P < 0.01 and ***P < 0.001.

We then continued to validate our hypothesis in in vivo mouse models. TTC staining results indicated an increased MI area in the I/R group compared to the control group (sham), while silencing TUG1 reduced the MI area o (Fig. 7A, B). H&E staining showed damaged cardiac structures and significant cell death in the I/R group, and the cardiac damage in the I/R + shTUG1 group was partially relieved compared to the I/R group (Fig. 7C). We also measured the serum markers for cardiac damage using ELISA, including serum LDH, creatine kinase (CK). and CK-MB. The results showed significantly elevated serum levels of these markers in I/R-treated mice (Fig. 7D–F). Further silencing of TUG1 significantly decreased the levels of cardiac damage markers, indicating protective effects. Similarly, TUNEL staining also showed increased cell death in cardiac tissue in I/R-treated mice, which was also inhibited by further silencing of TUG1 (Fig. 7G, H). These results show that silencing TUG1 reduces the cardiac damage caused by I/R in an in vivo model.

### FUS expression, mitochondrial dysfunction and pyroptosis were inhibited by silencing TUG1 in an in vivo mouse model

**Fig. 8:**
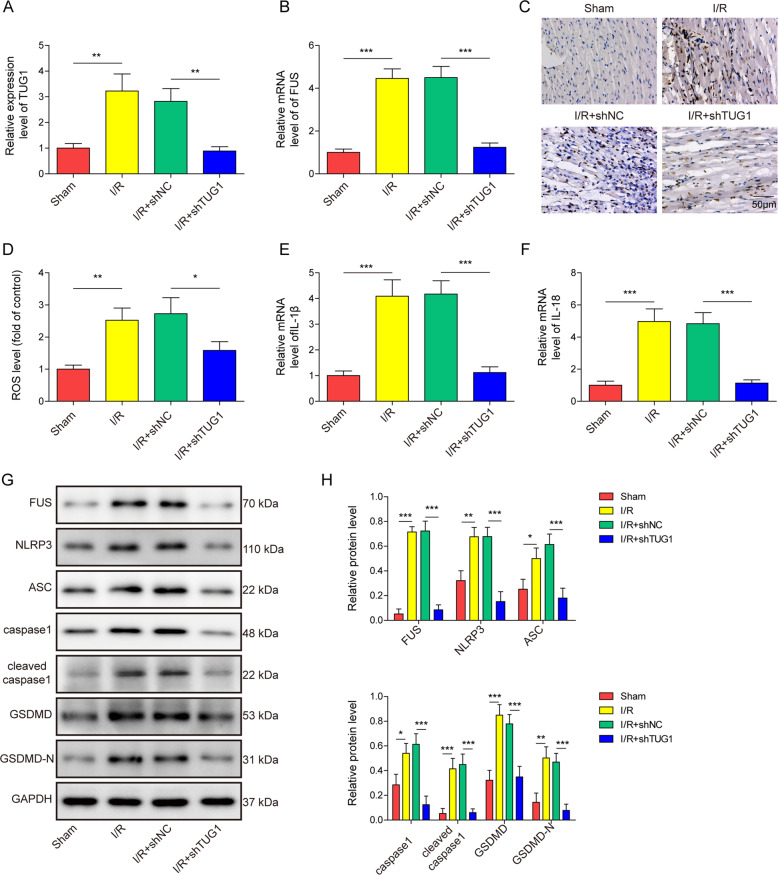
Silencing TUG1 reduced FUS expression and FUS-induced mitochondrial dysfunction and pyroptosis in a mouse model. The expression of **A** TUG1 and **B** FUS was measured using qRT-PCR in the myocardial tissue of I/R mice. **C** FUS expression in myocardial tissue was stained using immunohistochemistry. **D** ROS levels were measured using a DHE assay. The mRNA expression of **E** IL-1β and **F** IL-18 was measured using qRT-PCR. **G**, **H** The protein levels of FUS, NLRP3, ASC, and other downstream pyroptosis-related factors were measured using western blotting. *P < 0.05, **P < 0.01 and ***P < 0.001.

Further qRT-PCR analysis of the expression levels of TUG1 and FUS showed increased expression of these two factors in cardiac tissue in I/R-treated mice (Fig. 8A, B). FUS protein levels were also increased in cardiac tissue of I/R-treated mice, as shown in immunohistochemistry staining results (Fig. 8C). Further silencing of TUG1 inhibited the expression of FUS at both the mRNA and protein levels (Fig. 8A–C), indicating that TUG1 regulated FUS expression in an in vivo model. Serum ROS levels were increased by I/R treatment (Fig. 8D), which was inhibited by further silencing of TUG1. Levels of IL-1β and IL-18 were also increased by I/R treatment (Fig. 8E, F), accompanied by increased protein levels of FUS and pyroptosis-related markers, including NLRP3, ASC, caspase-1, GSDMD, cleaved caspase-1, and cleaved GSDMD (Fig. 8G, H), which also was inhibited by further silencing of TUG1, indicating that TUG1 also regulates the expression of downstream inflammatory factors and pyroptosis of cardiomyocytes.

## Discussion

MI is the most severe and fatal complication of ischemic heart disease, which causes millions of deaths globally every year [1]. Previous investigations suggested roles of pyroptosis and mitochondrial dysfunction in the pathogenesis of MI [5, 11], and HIF-1α and TUG1 were found to be involved in the process [12, 27]. However, the underlying pathway remains elusive for the roles of HIF-1α and TUG1 in MI. Our results showed that HIF-1α directly regulated TUG1 expression, which promoted pyroptosis and mitochondrial dysfunction of cardiomyocytes by binding to FUS and increasing its expression, and suppression of this pathway may help reduce the I/R-induced damage to heart tissue.

Although studies on HIF-1α in cardiovascular disease suggested a cardioprotective role [12], studies by Tang [13] and Shyu [14, 15] revealed that HIF-1α also contributed to I/R injury in MI or volume-overloaded heart failure. Our results, however, supported the detrimental role of HIF-1α in I/R injury in which HIF-1α contributed to the increase in TUG1 expression and subsequent mitochondrial dysfunction and pyroptosis. Similar roles of HIF-1α were shown in liver injury; inhibiting the activation of HIF-1α prevented mitochondrial dysfunction in I/R-induced injury to the liver [17], and ROS-induced nuclear translocation of HIF-1α promoted pyroptosis in myoblasts treated with hypoxia [18]. At the same time, other studies showed that HIF-1α inhibited pyroptosis of microglial cells after I/R injury [34]. These contradictory results may be explained by the different cells investigated (microglial cells [34] and cancer cells [35] versus liver cells [17], myoblasts [18], and cardiomyocytes in our study) and different treatment methods between those studies. Huang [34] and van Gisbergen [35] used oxygen deprivation to observe the effect of hypoxia in in vitro cell models, while studies by Bellanti [17], Yu [18], and our study used in vivo animal models treated with I/R or hypoxia-reoxygenation. In summary, the results of our study and previous findings by Tang [13] support a harmful role of HIF-1α in MI through increasing mitochondrial dysfunction and pyroptosis of cardiomyocytes. More investigations are required to further clarify the roles of HIF-1α in MI and other diseases.

Additionally, our results showed firstly that HIF-1α increased TUG1 expression through binding to its promoter region. A study by Cai [23] showed that TUG1 knockdown reduced hypoxia-reoxygenation-induced injury to cardiomyocytes via the miR-532-5p/Sox8 axis. Other studies by Li [25] and Yang [26] found that TUG1 overexpression promoted, while knockdown of TUG1 suppressed, apoptosis of cardiomyocytes. Our previous studies also suggested that silencing TUG1 expression reduced hypoxia-induced myocardial injury through regulation of ROS via the miR-132-3p/HDAC3 axis[27] or through regulation of autophagy [28]. Based on these findings, this study further showed that silencing TUG1 reduced hypoxia-induced myocardial injury by inhibiting mitochondrial dysfunction and pyroptosis. Additionally, a studyfound that lipopolysaccharide increased hypoxia/reoxygenation-induced injury to cardiomyocytes in a ROS-dependent manner [36]. Similarly, our results also suggested a role for ROS in promoting pyroptosis in cardiomyocytes. The results of those studies supported the roles of TUG1 in MI, possibly through mitochondrial dysfunction and pyroptosis mediated by increased ROS generation.

FUS, a DNA/RNA binding protein, has been intensively studied for its role in neurodegeneration, especially amyotrophic lateral sclerosis [37, 38]. Previous bioinformatics analysis found that FUS protein is one of the key proteins that regulate most of the differentially expressed genes in MI [39], and the binding between TUG1 and FUS was confirmed in our experimental results. In addition, a few studies reported possible roles of FUS in the pathogenesis of MI. Garikipati found that circular RNA circFndc3b promoted cardiac repair after MI by inhibiting the FUS/VEGF-A axis and apoptosis of cardiomyocytes [29]. A study by Wang reported that miR-200a regulated cardiomyocyte apoptosis by targeting FUS [40]. We found that FUS was involved in the HIF-1α/TUG1 signalling pathway, which contributed to mitochondrial dysfunction and cardiomyocyte pyroptosis in MI.

In conclusion, our study found that the HIF-1α/TUG1/FUS axis plays key roles in myocardial injury in MI by regulating mitochondrial dysfunction and cardiomyocyte pyroptosis, which potentially served as a treatment target for MI. Our study is the first to show that HIF-1α regulates TUG1 expression by directly binding to its promoter region, which may be the underlying mechanism of the detrimental roles of HIF-1α in MI.

## Materials and methods

### Cell culture and treatments

A mouse cardiomyocyte cell line (HL-1) was obtained from the American Type Culture Collection (ATCC, USA). Primary cardiomyocytes were collected from mice that were 1~4 days old. Briefly, mice were euthanized using carbon dioxide, and the heart was immediately collected, washed in D-Hanks solution (Sigma-Aldrich, USA) and minced using scissors. Minced heart tissue was digested using trypsin (prepared in D-Hanks solution, pH 7.4) at 37 °C for 20 min with gentle shaking every 2 min. Samples were then centrifuged at 1000 rpm for 5 min. The supernatant was discarded, and the cell pellet was washed with D-Hanks solution. Cells were resuspended in culture medium and transferred into flasks. The HL-1 cell line and isolated primary cardiomyocytes were cultured in DMEM/F12 culture medium (Gibco, USA) supplemented with 10% foetal bovine serum, 100 IU/ml penicillin, and 100 μg/ml streptomycin (Invitrogen, USA) in an incubator at 37 °C with 5% CO_2_ and 90% humidity. The cells were authenticated by STR profiling, and the cells were tested without contamination with mycoplasma. To mimic myocardial I/R injury in vitro, cardiomyocytes were subjected to hypoxia-reoxygenation (H/R) treatment. Briefly, cells were grown in glucose-free medium in an anaerobic chamber (95% N_2_ and 5% CO_2_) at 37 °C for 8 h. Then, the cells were returned to normal glucose-containing medium (4.5 mg/mL) and grown in a normal incubator with 95% air and 5% CO_2_ for another 12 h. Control cells were cultured in normal medium under normal culture conditions. For the inhibition of ROS, 3 mM N-acetyl-L-cysteine (NAC, Sigma-Aldrich) was used [41].

### Cell transfection

Short hairpin RNAs (shRNAs) targeting HIF-1α (shHIF-1α), TUG1 (shTUG1), FUS (shFUS), control shRNA (shNC), and full-length cDNA for HIF-1α or FUS were obtained from GenePharma (Shanghai, China) and transfected into HL-1 and primary mouse cardiomyocytes using the Adeno-X expression system (Clontech, USA) following the manufacturer’s instructions. HEK293 cells were used for the amplification of constructed vectors, and infection of target cells was performed at 100 plaque-forming units (PFU)/cell.

### CCK-8 assay

Cells were first cultured in a 96-well plate for 24 h. CCK-8 (SigmaAldrich, USA) was then added to each well at 1/10 of the volume of culture medium. The plate was incubated at 37 °C for 2 h. The results were obtained by measuring the absorbance at 450 nm using a plate reader (PerkinElmer, USA).

### Determination of ROS levels using dihydroethidium (DHE)

The determination of ROS levels was performed using a commercial ROS Detection Cell-Based Assay Kit (Cayman Chemical, USA) following the manufacturer’s instructions. Cells were cultured in a 96-well plate. After carefully removing the culture media, 150 μL of cell-based assay buffer was added to each well. The majority of the assay buffer was then carefully removed, leaving 10–20 μL of liquid at the bottom of the well. One hundred thirty microlitres of ROS staining buffer was added to each well. The plate was covered and incubated at 37 °C for 30 min in the dark. The staining buffer was carefully removed, and 100-μL cell-based assay buffer was added to each well. The results were obtained using a fluorescent plate reader (Thermo Fisher Scientific, USA) with excitation at 500 nm and emission at 590 nm.

### Determination of mitochondrial membrane potential

Mitochondrial membrane potential was determined using a commercial JC-1 mitochondrial membrane potential assay kit (ab113850, Abcam, USA) following the manufacturer’s instructions. Briefly, cells were seeded at 1.5 × 10^4^ cells per well in a dark 96-well plate and allowed to attach overnight. Cells were washed with 1x dilution buffer and incubated with 10 μg/mL JC-1 solution for 10 min at 37 °C in the dark. After washing the plate twice with 1x dilution buffer, the results were obtained under an Axio Imager A1 microscope (Carl Zeiss, Germany).

### Measurement of lactic acid dehydrogenase leakage

Measurement of LDH leakage was performed using a commercial LDH assay kit (ab65393, Abcam, USA). Briefly, 100-μL cells were seeded in a 96-well plate in triplicate. After incubation at 37 °C, the cells were centrifuged at 4 °C for 10 min, and the supernatant was transferred into a new 96-well plate. In each well, 100-μL LDH reaction mix was added, and the plate was incubated for 30 min at room temperature. The results were obtained using a microplate reader (Thermo Fisher Scientific, USA) with excitation at 450 nm and emission at 650 nm.

### Flow cytometry

Pyroptosis of cardiomyocytes was evaluated by measuring caspase-1 using flow cytometry. Briefly, a commercial FAM-FLICA in vitro Caspase Detection Kit (ImmunoChemistry, USA) was used following the manufacturer’s instructions. Samples were mixed with FLICA and PI and incubated at 37 °C for 1 h in the dark. After washing twice with wash buffer, the cells were analysed with a flow cytometer (BD Biosciences, USA).

### Chromatin immunoprecipitation

ChIP assays were performed using a SimpleChIP Enzymatic Chromatin IP Kit (#9003, Cell Signaling Technology, USA) following the manufacturer’s instructions. The protein-RNA interaction was first cross-linked using 1% formaldehyde. After cell lysis, chromatin was partially digested using micrococcal nuclease and sonication. The digested chromatin was then incubated with HIF-1α antibody (#36169, Cell Signaling Technology, USA) or acetyl-histone H3 antibody (#9649, Cell Signaling Technology, USA) overnight at 4 °C. After pulldown by ChIP-Grade Protein G Magnetic Beads (#9006, Cell Signaling Technology, USA), the immune complex was eluted from the beads, and DNA was purified. The presence of TUG1 was examined using quantitative real-time PCR (qRT-PCR) with the primer pair forwards 5′-GGCACCCAGTGTAAAGCA-3′ and reverse 5′-AAGCAGCAGATAACAGAGTTGA-3′ (GenePharma, Shanghai, China). Rabbit immunoglobulin G (IgG) (#8726, Cell Signaling Technology, USA) was used as a negative control. PCR products were then separated using gel electrophoresis.

### Luciferase reporter assay

The JASPAR database (http://jaspar.genereg.net/) was used to predict the potential binding sequence of HIF-1α and the TUG1 promoter. Following the analysis results, the TUG1 promoter sequence where HIF-1α binds was cloned into the pGL3 vector (Promega, USA). The vector was transfected into HL-1 and primary cardiomyocytes using Lipofectamine 2000 (Thermo Fisher Scientific, USA), which were then cotransfected with adenovirus vector carrying hHIF-1α, shNC, HIF-1α or empty vector following the manufacturer’s instructions. After transfection for 48 h, cells were harvested, and luciferase activities were detected by the Dual-Luciferase Reporter Assay Kit (Promega). The ratio of firefly activity to Renilla luciferase activity was subsequently determined.

### RNA immunoprecipitation

RNA immunoprecipitation (RIP) was performed using a Magna RIP RNA-Binding Protein Immunoprecipitation Kit (Millipore, USA). HL-1 and primary mouse cardiomyocytes were cultured in flasks until ~80% confluence. After washing with ice-cold PBS, the cells were scraped and harvested by centrifugation at 1500 rpm for 5 min at 4 °C. After lysis in complete RIP lysis buffer, the cell lysate was centrifuged at 14,000 rpm for 10 min at 4 °C, and the supernatant was transferred to a new tube containing RIP immunoprecipitation buffer and magnetic beads bound with antiFUS antibody (ab245332, Abcam, USA) or rabbit IgG (ab172730, Abcam, USA). After incubation with gentle rotation overnight at 4 °C, the samples were placed on a magnetic separator (Grainger, USA), and the supernatant was discarded. The beads were washed six times with cold RIP Wash Buffer. Protein K buffer was added, and the mixture was incubated at 55 °C for 30 min to digest the proteins. After purification of RNA, qRT-PCR was used to detect TUG1 in the samples.

### Separation of mitochondrial proteins

The separation of mitochondrial proteins was performed using Mitochondrial Protein Isolation Buffer (#97063-126, VWR Life Science, USA) following the manufacturer’s instructions. Briefly, 1 × 10^6^ cells were resuspended in 400-μL Mitochondrial Protein Isolation Buffer and homogenized 20 times on ice. After centrifugation, supernatant containing cytosolic proteins was transferred into a new tube, and the remaining pellet was resuspended in 1 mL Mitochondrial Protein Isolation Buffer and centrifuged. The pellet containing mitochondrial proteins was resuspended in 40-μL Mitochondrial Protein Isolation Buffer and frozen for future analysis.

### Construction of the in vivo I/R mouse model

An in vivo mouse MI model was constructed using 10-week-old male C57BL/6 mice as described previously [27]. In all, 4 groups of animals (8 mice per group, which were chosen based on our previous studies and pilot experiments) were used, including I/R-treated mice (I/R), I/R-treated mice transfected with shTUG1 (I/R + shTUG1), I/R-treated mice transfected with shNC (I/R + shNC), and control animals (sham). Animals were purchased from Shanghai SLAC Experiment Animal Co. (Shanghai, China), randomly allocated into the 4 groups and kept in an SPF-grade animal-holding facility with a 12-h light:dark cycle. To generate a TUG1-silenced animal model, the aorta and pulmonary arteries were briefly cross-clamped (for 20 sec), and an adenovirus vector carrying shTUG1 or shNC was quickly injected into the aortic root within the time period. After 5 days of recovery, to generate the I/R mouse model, the left anterior descending artery of the animals was ligated for 1 h to induce ischemia in the heart tissue and then reopened to allow reperfusion for 24 h. Animals in the negative control group received sham operation instead. Based on pre-established criteria, animals that did not survive the above procedures were excluded from further analysis. Animals were then euthanized using carbon dioxide, and the heart was quickly collected and processed for sectioning and subsequent staining. The investigators who performed the experiments were blinded to the group allocation. All animal experiments and protocols were reviewed and approved by the Animal Care and Use Committee of Guilin Medical University.

### TTC staining

Before TTC staining, the heart samples were first frozen and sliced into 1-mm-thick sections. The sections were then stained with 1% 2,3,5-triphenyltetrazolium chloride (TTC) solution (Sigma–Aldrich, USA) for 15 min at 37 °C. The stained slides were photographed under a light microscope (Leica Microsystems, USA) and analysed using ImageJ software. The percentage of MI area was calculated by the area of infarction divided by the total area of the section.

### H&E staining

For H&E staining, heart samples were first fixed in 10% formalin and embedded in paraffin. The sample blocks were sectioned into 4-μm-thick sections, deparaffinized in xylene, and rehydrated in 100%, 95%, and 80% ethanol. The slides were then stained with haematoxylin for 3 min and washed three times under deionized water for 30 s. After differentiation in 1% acid ethanol, slides were stained with 0.5% eosin for 30 s, dehydrated in ethanol and xylene, and sealed in mounting medium with coverslip. After drying overnight at room temperature, the slides were examined and photographed under a light microscope (Leica Microsystems, USA).

### TUNEL staining

A TUNEL Apoptosis Detection Kit (FITC) was purchased from Sangon Biotech (#E607178, Shanghai, China), and TUNEL staining was performed following the manufacturer’s instructions. Sample sections were treated with Protein K working solution for 10 min at 37 °C. After incubation with the reaction mixture for 2 h at 37 °C, Blocking Reagent was added, and the samples were incubated for 30 min at room temperature. Samples were then incubated with biotin-conjugated anti-digoxin antibody and SABC-FITC. After counterstaining with diamidino-2-phenylindole (DAPI), slides were mounted with mounting medium and observed under a fluorescence microscope (Leica Microsystems, USA)

### Immunohistochemistry

Sample blocks were first sectioned at 4-μm thickness, deparaffinized in xylene, and rehydrated in ethanol and deionized water. After heat-mediated antigen retrieval, slides were stained with antiFUS antibody (1:100, ab124923, Abcam, USA) overnight at 4 °C. The next day, the slides were washed with 1x TBS-T and stained with HRP-conjugated goat antirabbit IgG secondary antibody (1:5000, ab205718, Abcam, USA) for 1 h. After counterstaining using haematoxylin for 30 s, the slides were washed and stained with 3,3′-diaminobenzidine. The results were examined and photographed under a light microscope (Leica Microsystems, USA).

### Measurement of serum LDH, creatine kinase, and CK-MB using ELISA

Enzyme-linked immunoassay (ELISA) kits for CK (ab155901, Abcam, USA), LDH (A020-2-2, Nanjing JianCheng, China), and CK-MB (E006-1-1, Nanjing JianCheng, China) were used to measure their serum levels in an in vivo MI mouse model following the manufacturer’s instructions.

### Extraction of total RNA, reverse transcription, and qRT-PCR

Total RNA was extracted from cells or tissue samples using TRIzol reagent (Invitrogen, USA) following the manufacturer’s instructions. cDNA was synthesized by reverse transcription using a TaqMan MicroRNA Reverse Transcription kit (Invitrogen, USA). qRT-PCR was then performed on an Applied Biosystems 7500 Real Time PCR System (Thermo Fisher Scientific, USA) using a reaction mixture consisting of 10 μL SYBR-Green Universal qPCR Master Mix (Bio-Rad, USA). β-Actin was used as an internal reference for mRNA. Specific primers for qRT-PCR are listed below: IL-1β, forwards 5′-TGGACCTTCCAGGATGAGGACA-3′, reverse 5′-GTTCATCTCGGAGCCTGTAGTG-3′; IL-18, forwards 5′-GACAGCCTGTGTTCGAGGATATG-3′, reverse 5′-TGTTCTTACAGGAGAGGGTAGAC-3′; TUG1, forwards 5′-GGCACCCAGTGTAAAGCA-3′, reverse 5′-AAGCAGCAGATAACAGAGTTGA-3′; FUS, forwards 5′-GCTACTCCCAACAGAGCAGTCA-3′, reverse 5′-GAGCTGACTGAGTTCCATAGCC-3′; β-actin, forwards 5′-CATTGCTGACAGGATGCAGAAGG-3′, reverse 5′-TGCTGGAAGGTGGACAGTGAGG-3′.

### Western blot

Total cell proteins were extracted from cardiomyocytes or heart tissue using radioimmunoprecipitation assay (RIPA) buffer (Thermo Fisher Scientific, USA). Mitochondrial proteins were extracted as described in the previous section. The protein concentration of the samples was determined using a bicinchoninic acid (BCA) protein assay kit (Thermo Fisher Scientific, USA). Equal amounts of proteins were first separated on sodium dodecyl sulfate-polyacrylamide gel electrophoreses (SDS-PAGE) gels using electrophoresis and then transferred to polyvinylidene difluoride membranes. After blocking with 5% skimmed milk dissolved in TBS-T for 1 h at room temperature, membranes were washed in TBS-T and incubated at 4 °C overnight with primary antibodies (all from Cell Signaling Technology, unless otherwise stated): NLRP3 (#15101, 1:1000), ASC (#67824, 1:1000), caspase-1 (#24232, 1:1000), cleaved caspase-1 (#89332, 1:1000), GSDMD (ab219800, 1:1000, Abcam, USA), cleaved GSDMD (#10137, 1:1000), GAPDH (#5174, 1:1000), HIF-1α (#36169, 1:1000), FUS (#4885, 1:1000), or COX IV (#11967, 1:1000). After incubation, membranes were washed with tris-buffered saline with Tween (TBS-T) and incubated with corresponding secondary antibodies: HRP-linked antimouse IgG antibody (#7076, Cell Signaling Technology, USA) or HRP-linked antirabbit IgG antibody (#7074, Cell Signaling Technology, USA) for 2 h at room temperature. Signals were developed using an enhanced chemiluminescence (ECL) system (Beyotime, China), and ImageJ was used to analyse the density of bands.

### Statistical analysis

All experiments were repeated at least three times, and the results are summarized as the mean ± standard deviation (SD). Statistical analysis was performed using GraphPad Prism 6 (GraphPad Software, USA). All data were normally distributed, and variance was similar between the groups that were statistically compared. *Student’s t*-test was used for comparisons between two groups, while one-way analysis of variance (ANOVA) followed by Tukey’s post-*hoc* test was used for comparisons between multiple groups. The result was considered statistically significant if *P* < 0.05 (two-sided).

## Supplementary information

Revised Manuscript-Marked Up

Language Editing Certificate

author contribution form

## Data Availability

All data generated or analyzed during this study are included in this article. The datasets used and/or analyzed during the current study are available from the corresponding author on reasonable request.
