# Supplementary figures and images for: HIF-1α-regulated lncRNA-TUG1 promotes mitochondrial dysfunction and pyroptosis by directly binding to FUS in myocardial infarction
Source: Cell Death Discov. 2022 Apr 8;8:178. doi: 10.1038/s41420-022-00969-8 (PMC8993815; doi:10.1038/s41420-022-00969-8)

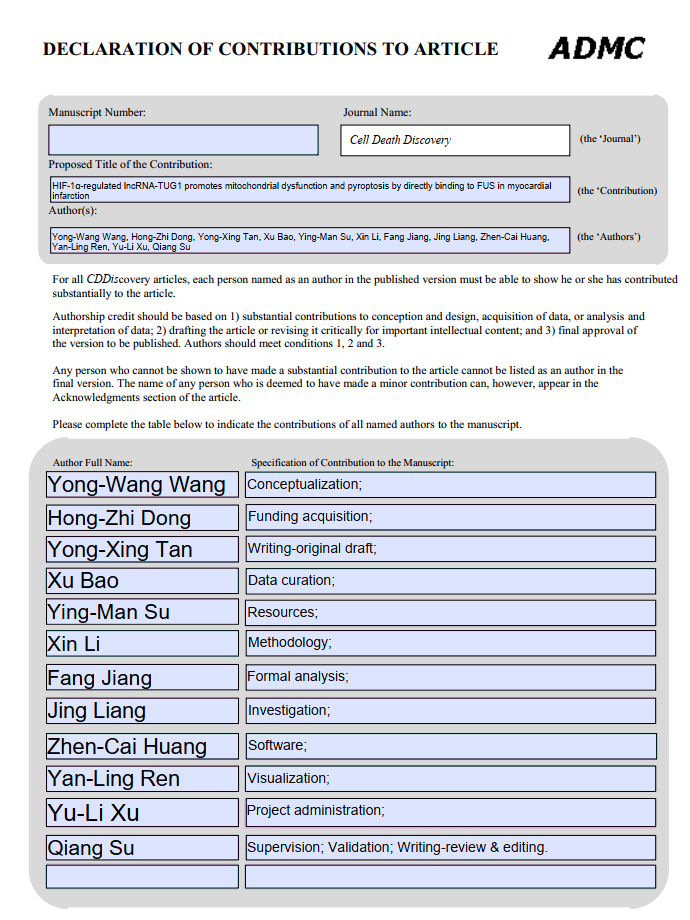


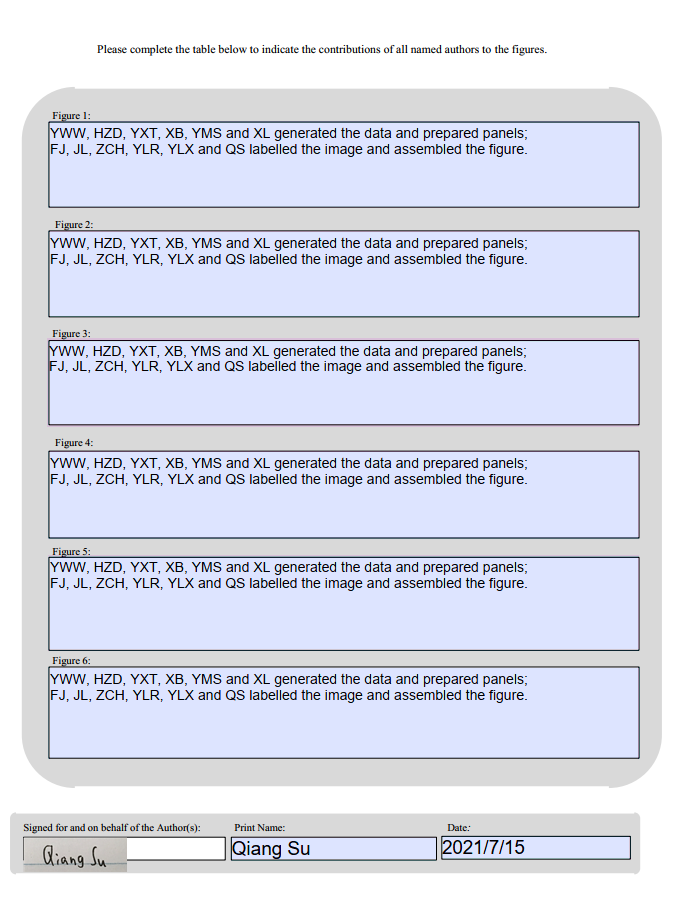

Supplement: Supplementary file 3 — author contribution form [file 41420_2022_969_MOESM3_ESM.docx]
